# Paranoia in patients attending child and adolescent mental health services

**DOI:** 10.1177/0004867420981416

**Published:** 2021-01-10

**Authors:** Jessica C Bird, Emma C Fergusson, Miriam Kirkham, Christina Shearn, Ashley-Louise Teale, Lydia Carr, Hannah J Stratford, Antony C James, Felicity Waite, Daniel Freeman

**Affiliations:** 1 Oxford Cognitive Approaches to Psychosis, Department of Psychiatry, University of Oxford, Oxford, UK; 2 Oxford Health NHS Foundation Trust, Oxford, UK

**Keywords:** Youth mental health, psychotic experiences, delusions, emotional disorders, network analysis

## Abstract

**Objective:**

Paranoia may be particularly prevalent during adolescence, building on the heightened social vulnerabilities at this age. Excessive mistrust may be corrosive for adolescent social relationships, especially in the context of mental health disorders. We set out to examine the prevalence, symptom associations, and persistence of paranoia in a cohort of young people attending child and adolescent mental health services.

**Method:**

A total of 301 patients (11–17 years old) completed measures of paranoia, affect, peer difficulties and behavioural problems. Clinicians also rated each participant’s psychiatric symptoms. Patterns of association were examined using linear regressions and network analyses. In total, 105 patients repeated the measures several months later.

**Results:**

Most of the adolescents had affective disorders (*n* = 195), self-harm/suicidality (*n* = 82), or neurodevelopmental conditions (*n* = 125). Few had suspected psychosis (*n* = 7). Rates of paranoia were approximately double compared with previous reports from the general population. In this patient sample, 35% had at least elevated paranoia, 15% had at least moderate paranoia, and 6% had high paranoia. Paranoia had moderate associations with clinician-rated peer difficulties, self-harm, and trauma, and small associations with clinician-rated social anxiety, depression, generalised anxiety, and educational problems. Network analyses showed paranoia had the strongest unique relationship with peer difficulties. Paths from peer difficulties to anxiety, self-harm, post-traumatic stress disorder symptoms, and behavioural problems were all via paranoia. Both self-harm and post-traumatic stress disorder were solely associated with paranoia in the network. Paranoia remained persistent for three-quarters and was associated with greater psychological problems over time.

**Conclusion:**

Paranoia is relatively common and persistent across a range of clinical presentations in youth. When paranoia occurs alongside emotional problems, important peer interactions may be adversely affected. Wider consideration of paranoia in adolescent patients is needed.

## Introduction

Paranoia – the unfounded idea that others deliberately intend harm – is one of the most prominent symptoms of psychotic disorders. Yet the clinical reality is that paranoia is rarely specific to psychosis, with evidence it occurs across a range of disorders (D’Agostino et al., 2019; Freeman et al., 2019a). Indeed, there is growing evidence that paranoia builds upon concerns about the self (e.g. social vulnerability, low self-esteem) and psychological processes (e.g. threat anticipation, worry) central to many emotional disorders (Freeman, 2016). In adolescence, an age when feelings of social vulnerability are typically heightened, paranoia may be especially prevalent (Bird et al., 2019; Ronald et al., 2014). Paranoia in adolescents is associated with a range of psychological difficulties including affective symptoms, peer difficulties, behavioural problems, and poor sleep (Bird et al., 2019; Taylor et al., 2015; Wigman et al., 2011; Zavos et al., 2014). Persistent paranoia has the potential to leave young people feeling unsafe in their daily lives, mistrustful in relationships, and isolated. The resulting effects on social relationships during this sensitive period for social interaction (Orben et al., 2020) could have far-reaching impact, with evidence that poor social functioning predicts the long-term persistence of psychiatric disorders in adolescence (Ford et al., 2017). To date, however, there has been extremely little detailed research on paranoia in clinical populations of youth.

There is a substantial literature showing psychotic experiences in general are common in adolescents accessing services, and, although transient for a number, the presence of such symptoms indicates a pluripotent risk for multiple psychiatric disorders and poor outcomes (Kelleher et al., 2012; McGorry et al., 2018). However, individual psychotic experiences such as paranoia, hallucinations, grandiosity, and cognitive disorganisation are separable phenomenon (found to be distinct in factor analytic studies) (e.g. Peralta and Cuesta, 1999; Ronald et al., 2014) that can occur independently of each other (e.g. Hermans et al., 2020) and that have a degree of aetiological difference (e.g. Garety et al., 2013; Zavos et al., 2014). Individual psychotic experiences will require a degree of difference in explanation and tailoring of treatment. The effects on day-to-day life may also vary – social relationships, for example, may be especially affected by paranoia due to the mistrust of others inherent in the cognitions.

In recent years, significant advances have been made in the treatment of persecutory delusions in adults by adopting a targeted focus on paranoia and its contributory causal factors (Freeman, 2016). Yet much of the adolescent literature has conceptualised psychotic experiences as a single construct, with individual symptoms primarily viewed as interchangeable indicators of psychosis risk. As a result, studies typically include measures that sum together a broad range of psychotic experiences into a total score, with individual domains often assessed to unequal degrees. Indeed, these measures typically include only one or two items for each psychotic experience, and, so, may have limited precision for detecting (and understanding) those symptoms. Much of the adolescent literature is also biased towards the assessment of hallucinations, which is often the only consistently defined construct across different measurement tools, and in many instances is used as a proxy for all psychotic experiences (e.g. Kelleher et al., 2017).

Here, we adopt a targeted approach: systematically assessing paranoia and potential correlates in a cohort of adolescents accessing UK Child and Adolescent Mental Health Services (CAMHS). We had three objectives. The first objective was to describe the prevalence of paranoia in this cohort using a measure specifically validated for adolescents and compare these rates to previous reports from the general population (Bird et al., 2019). The second objective was to examine the patterns of association between paranoia, psychiatric symptoms, and social functioning. To do this, the bivariate associations between paranoia and the presence of clinician-rated symptoms were first examined; then, network analysis was used to examine the unique relations with self-report and selected clinician-rated symptoms. Network approaches can statistically estimate complex systems of interaction (Borsboom and Cramer, 2013), therefore providing potential insights into the mechanisms linking paranoia with other difficulties. The final objective was to examine the persistence of paranoia in a subgroup of the cohort and its relationship with other difficulties over time.

## Method

### Participants

Over 15 months, adolescents (11–17 years) were recruited during routine clinical appointments at a Tier 3 outpatient CAMHS team and a Tier 4 adolescent inpatient unit based in Oxfordshire, UK. Both services were part of Oxford Health National Health Service (NHS) Foundation Trust. In the United Kingdom, Tier 3 CAMHS provide specialist multidisciplinary assessment and treatment for adolescents under 18 years with complex mental health problems and Tier 4 units provide highly specialist care for under 18s requiring admission for severe psychiatric problems and high levels of risk. Participants were invited to take part regardless of their reason for accessing services, clinical diagnosis, or current treatment. The only exclusions were a moderate/severe learning disability or inability to complete questionnaires in English. Informed parental consent and child assent (11–15 years) or consent (16–17 years) was obtained prior to taking part. The study received approval by an NHS Research Ethics Committee (Ref: 17/SC/0539).

### Measures

#### The Bird Checklist of Adolescent Paranoia

The Bird Checklist of Adolescent Paranoia (B-CAP; Bird et al., 2019, 2020) is an 18-item self-report scale for adolescents that assesses the frequency of paranoid thoughts in the past fortnight. Items are rated on a 6-point scale (0 = never, 5 = all the time) with higher scores indicating higher paranoia. Three subtypes of paranoia are assessed within an overarching single construct: social harm, conspiracy ideas, and physical threat. The B-CAP has very good psychometric properties including strong reliability across the severity spectrum and measurement invariance for both age and gender in adolescents (Bird et al., 2020). The B-CAP also demonstrates good concurrent validity with other measures of paranoia and adolescent’s reports that their fears of others are excessive (Bird et al., 2019). We recently validated score ranges for the B-CAP where a score of 23+ indicates mildly elevated paranoia, 40+ indicates moderate paranoia, 54+ indicates high paranoia, and 71+ indicates severe paranoia (Bird et al., 2020).

#### The Revised Child Anxiety and Depression Scale

The Revised Child Anxiety and Depression Scale (RCADS; Chorpita et al., 2000) is a 47-item self-report questionnaire examining anxiety and depression in 8- to 17-year olds. Items are rated on a 4-point scale (0 = never, 3 = always) with higher scores indicating higher severity. Six subscales are produced: depression, panic, obsessive compulsiveness, generalised anxiety, social anxiety, and separation anxiety.

#### The Strengths and Difficulties Questionnaire

The Strengths and Difficulties Questionnaire (SDQ; Goodman, 2001) is a 25-item mental health screening questionnaire for adolescents aged 11–17 years. Items are rated on a 3-point scale (0 = not true, 2 = certainly true), with higher scores indicating greater difficulties. Four problem subscales are derived comprising emotional symptoms, conduct problems, hyperactivity/inattention, and peer difficulties. An additional ‘impact’ score is derived from items concerning overall distress and social impairment (Goodman, 1999). The emotional symptoms domain was not included in the analysis due to the conceptual overlap with the RCADS.

#### The Current View

The Current View (Jones et al., 2013) is a practitioner-completed tool assessing a wide range of clinical difficulties. Here, we examined clinician ratings of the following psychiatric symptoms and indicators of social functioning: anxiety (separation, social, generalised, obsessive-compulsive disorder [OCD], panic, and agoraphobia), depression, deliberate self-harm, fluctuations in mood (bipolar), hallucinations/delusions (psychosis), post-traumatic stress disorder symptoms, substance abuse, conduct problems, emerging personality disorder, attention-deficit hyperactivity disorder (ADHD), autism spectrum disorder (ASD), history of abuse/neglect, peer relationship problems, persistent family relationship problems, and current educational problems. All items were coded to indicate presence/absence of that problem, except for educational difficulties where the sum of two items rating severity of attendance and attainment problems on a 3-point scale was used.

### Procedure

Participants completed the paranoia questionnaire alongside the routinely administered RCADS and SDQ. Clinicians involved in each participant’s care (i.e. care coordinator or psychiatrist) completed a routine measure of current difficulties (i.e. Current View). All three routine measures were completed as part of participant’s standard care. Case note diagnoses/presenting problems were obtained from the diagnosis section of participant’s electronic records, recent clinical assessment/review letters, and discussion with care coordinators. The study involved an optional follow-up where the self-report questionnaires were repeated after at least 3 months for a subsample of participants who were contactable and agreed to do so. Follow-up questionnaires were completed at the clinic or online via a Qualtrics survey.

### Statistical analysis

All analyses were conducted in R, version 3.6.1 (R Core Team, 2013). For each questionnaire, missing values were imputed using predictive mean matching for individuals with missing data for less than 20% of items. As the Current View items were examined individually as distinct variables, missing values were not imputed.

#### Prevalence

Paranoia prevalence was assessed with mean scores, item endorsement defined as a score of 2+ (i.e. ‘couple of times’ in past 2 weeks), and the proportion scoring above validated B-CAP thresholds (Bird et al., 2020). Paranoia scores were compared between genders using a *t*-test and the correlation between paranoia and age was examined.

Prevalence rates of paranoia in this sample were presented alongside previously reported mean scores and item endorsements on the B-CAP from a representative dataset of 801 adolescents aged 11–15 years (mean age = 13.3, standard deviation [SD] = 1.16, girls = 410, boys = 382, other gender = 9) from a secondary school in the United Kingdom (Bird et al., 2019). Here, we report the proportion of adolescents from this school cohort who scored above recently validated B-CAP score ranges (Bird et al., 2020) to enable direct comparison with the clinical sample.

#### Clinical associations

The bivariate relationships between paranoia and the presence of clinician-rated difficulties were assessed using a series of linear regressions. We did not correct for non-normality in the residuals as linear regression models without normally distributed errors produce valid estimates in large samples (Schmidt and Finan, 2018). For eight variables, however, weighted least squares (WLS) regression was used to account for heteroscedasticity in the residuals (Romano and Wolf, 2017). Standardised beta (β) estimates are presented with 95% confidence intervals (CIs).

Network analysis was used to estimate the unique patterns of association between paranoia, self-report psychological problems, and the clinician-rated presence of two distinct symptoms with clinical relevance to paranoia: deliberate self-harm and post-traumatic stress. In a network model, individual variables are represented by *nodes*, and pairs of nodes may be connected by an *edge* that indicates the presence of an association after conditioning on all other variables (Borsboom and Cramer, 2013). Consequently, the lack of an edge between two variables indicates an absence of a relationship once all other variables are known.

Due to the mixture of continuous and binary variables in our data, we estimated a Mixed Graphical Model (MGM) using the package ‘mgm’ (Haslbeck and Waldorp, 2020). Missing data was handled using listwise deletion, resulting in a sample of 218 participants with complete data on all 13 variables. To overcome potential sampling variation and limit the estimation of spurious edges, we used a regularisation technique with the Least Absolute Shrinkage and Selection Operator (LASSO; Tibshirani, 1996). The LASSO regularisation employs a penalty by limiting the sum of the partial correlation coefficients, leading to a shrinking of estimates with some becoming exactly zero (Epskamp and Fried, 2018). The degree of regularisation is controlled by the tuning parameter λ, selected using the extended Bayesian information criterion (EBIC). The EBIC hyperparameter is set between 0 and 0.5 to determine the extent to which a parsimonious model is preferred (Foygel and Drton, 2010), with higher values producing more cautious estimations. We used an EBIC hyperparameter of 0.3. Node predictability was also estimated to show the extent to which each node is predicted by its neighbouring nodes (i.e. those it shares an edge with); this represents the proportion of variance explained (*R*^2^) for continuous variables and the proportion of correct classification (CC_total_), or accuracy, for binary variables (Haslbeck and Waldorp, 2020). We also calculated the normalised accuracy (nCC) for binary variables which break down the CC_total_ to represent the additional contribution of connected nodes beyond what can be trivially predicted from the marginal intercept model (CC_marg_) (Haslbeck and Waldorp, 2018).

Once estimated, the unique relations among the variables were visualised using the package ‘qgraph’ (Epskamp et al., 2012) in a weighted network model where the thickness and saturation of the edge colour represents the size of the relationship. Blue edges represent positive conditional dependence associations while red edges represent negative associations. The node predictability values are visualised by a shaded ring around each node. For the binary variables, these rings are split to represent the accuracy of the intercept model and the additional contribution of connected nodes. No minimum edge weight was set in the visualisation. The network layout was determined by the Fruchterman and Reingold (1991) algorithm, positioning the most strongly connected nodes in the centre. In a separate graph, the shortest paths between paranoia and every other variable were computed to highlight potential mediation pathways in the network. Calculated using Dijkstra’s (1959) algorithm, the shortest path represents the fastest route to get from one node to another, taking the strength of edge weights along different possible routes into account. Edges not required for the shortest paths are suppressed, allowing a clear visualisation of the direct and indirect pathways between selected variables.

For all edges, 95% CIs were constructed using a non-parametric bootstrap with 1000 iterations in the package ‘bootnet’ (Epskamp et al., 2018). The bootstrap difference test was used to compare edge weights. Due to the regularisation, edge weights are biased towards zero and thus CIs cannot be interpreted as a significance test against zero (Epskamp et al., 2018).

#### Paranoia persistence

Follow-up data were collected for paranoia and the two other self-report measures in a subgroup of participants. Change in paranoia over time was examined using the effect size (ES) formula = *M*_pre_-*M*_post_/SD_pre_ and a Wilcoxon signed-rank test. Individual change in paranoia was examined using the reliable change index (RCI; Jacobson and Truax, 1991) where an RCI of ±1.96 indicates significant change. For the RCI calculation, the B-CAP Cronbach’s α of 0.94 from the current sample was used. To examine the relationship between paranoia persistence and symptoms over time, participants were split into a persistent/increasing paranoia group (⩾23 at both times, or ⩾23 at either time point with non-significant RCI) and a low/transient paranoia group (⩽22 at both times, or significant decreases to ⩽22 at follow-up). Using the package ‘lme4’ (Bates et al., 2015), linear mixed-effects models were conducted for each symptom domain with fixed effects for paranoia group, time, and a group by time interaction, and a random effect for participants.

## Results

### Participant characteristics

A total of 301 adolescents took part (mean age = 15.1, SD = 1.75). There was a higher proportion of girls (*n* = 184, 61%) than boys (*n* = 117, 39%) and most were White British (*n* = 240, 80%). Participants included 271 community CAMHS patients (mean age = 15.0, SD = 1.80, girls = 164, boys = 107) and 30 inpatients (mean age = 16.0, SD = 0.81, girls: *n* = 20, boys: *n* = 10). Adolescents were accessing services with a range of problems, although the most common were affective disturbances and neurodevelopmental conditions (Table 1). Seven participants had suspected psychosis and an additional four were noted to experience hallucinations alongside other difficulties. Beyond those who had suspected psychosis, paranoia was recorded as a presenting problem in the clinical records of only one participant.

**Table 1.** Primary presenting problem(s) for accessing CAMHS as recorded by participant’s care team and mean paranoia scores for each problem.

	*n*	Percentage	Paranoia (SD)
Anxiety/depression	195	65	22.0 (19.8)
Emotion dysregulation, self-harm and suicidality	82	27	27.4 (19.5)
Autism spectrum disorder	79	26	21.4 (21.2)
Attention-deficit hyperactivity disorder	41	14	12.7 (13.2)
Anger/conduct problems	30	10	17.3 (16.7)
Disordered eating	24	8.0	21.2 (18.6)
Trauma	23	7.6	25.5 (19.7)
Sleep problems	20	6.6	21.6 (16.3)
Gender identity issues	8	2.7	19.2 (18.7)
Family relationship issues	8	2.7	17.8 (13.5)
Psychosis	7	2.3	26.1 (23.9)
Substance misuse	7	2.3	23.9 (17.4)
Tic disorders	5	1.7	19.8 (30.1)
Hallucinations^a^	4	1.3	23.8 (22.6)
Paranoia^a^	1	0.3	32.0 (NA)

SD: standard deviation; NA: not applicable; CAMHS: Child and Adolescent Mental Health Services.

a Occurring alongside other difficulties in participants without suspected psychosis.

### Prevalence

Paranoid thoughts were common in this clinical sample, with item endorsement ranging from 14% to 54% (Table 2). The mean number of suspicions endorsed was 5.85 (SD = 5.17). Out of the 301 patients, 35% had at least mildly elevated paranoia, 15% had at least moderate paranoia, 6% had at least high paranoia, and 3% had severe levels of paranoia (Table 3). As shown in Tables 2 and 3, the rates of paranoia were approximately double those previously reported in a general population sample of adolescents.

**Table 2.** B-CAP item endorsement in CAMHS sample (*n* = 301) and previously reported weekly rates from the general population (*n* = 801).

	CAMHS							Non-clinical^a^
Item	0	1	2	3	4	5	Weekly+	Weekly+
1. People at school are trying to make me feel unwanted	135	33	68	37	17	11	44%	25%
2. I’m sure people are gossiping about me on social media	120	39	76	31	12	23	47%	21%
3. I am being pushed out of conversations on purpose	124	54	63	28	22	10	41%	22%
4. My friends or partner are ignoring my messages to upset me	177	49	32	21	12	10	25%	10%
5. People are trying to embarrass me in class on purpose	185	39	31	24	9	13	26%	20%
6. People are making sly comments to upset me	118	58	60	36	14	15	42%	16%
7. I think people are lying to me on purpose	93	44	74	47	19	24	54%	30%
8. People say things under their breath to wind me up	143	43	48	33	18	16	38%	24%
9. Nasty tricks are being played on me	216	32	30	14	1	8	18%	8%
10. People are trying to confuse me on purpose	164	40	48	19	15	15	32%	17%
11. Groups of people are planning against me	197	35	31	17	11	10	23%	10%
12. People are collecting my information or photos to use against me	237	21	23	7	4	9	14%	7%
13. I’m sure people are seeking revenge on me	201	36	30	17	8	9	21%	11%
14. I feel like I am being followed or stalked	212	23	26	18	9	13	22%	12%
15. I am scared of what strangers will do to me	124	50	45	35	22	25	42%	32%
16. People will try to kidnap me	193	42	26	22	11	7	22%	14%
17. I could be attacked at any time	132	54	43	21	25	26	38%	23%
18. I feel unsafe around people everywhere I go	149	46	37	20	23	26	35%	19%

CAMHS: Child and Adolescent Mental Health Services; B-CAP: Bird Checklist of Adolescent Paranoia.

a Endorsement rates as reported in Bird et al. (2019).

**Table 3.** Mean scores and proportions of CAMHS patients (*n* = 301) scoring above validated score thresholds compared to previously collected data from the adolescent general population (*n* = 801; Bird et al., 2019).

	CAMHS			General population		
	All	Girls	Boys	All^a^	Girls	Boys
Mean score (SD)	20.0 (18.2)	23.1 (19.4)	15.0 (14.9)	12.5 (14.0)	15.8 (15.0)	8.2 (10.8)
⩽22 (average range)	197 (65%)	108 (59%)	89 (76%)	667 (83%)	314 (77%)	351 (92%)
23+ (mildly elevated+)	104 (35%)	76 (41%)	28 (24%)	134 (17%)	96 (23%)	31 (8%)
40+ (moderate+)	46 (15%)	34 (18%)	12 (10%)	52 (7%)	40 (10%)	8 (2%)
54+ (high+)	18 (6%)	15 (8%)	3 (3%)	16 (2%)	11 (3%)	3 (0.8%)
71+ (severe+)	10 (3%)	9 (5%)	1 (0.9%)	4 (0.5%)	2 (0.5%)	2 (0.5%)

CAMHS: Child and Adolescent Mental Health Services; SD: standard deviation.

a In the general population sample, 9/801 participants identified as ‘other gender’. These participants were not included in the gender group comparison due to the limited sample size.

Paranoia in the patient sample was significantly higher in girls than boys (*t* = 4.08, *df* = 288.2, *p* < 0.001), with 41% of girls reporting at least mildly elevated levels compared to 24% of boys. There was no relationship between age and paranoia (*r* = 0.08, *p* = 0.16). The 30 inpatients had somewhat higher paranoia scores overall (mean = 27.1, SD = 21.5) than the community patients (mean = 19.2, SD = 17.7), although this was not significant (*t* = 1.93, *df* = 33.5, *p* = 0.062).

### Clinical associations

The clinician-rated Current View was completed for 272 participants (mean age = 15.0, SD = 1.77, girls: *n* = 166, boys: *n* = 106, outpatient: *n* = 248, inpatient: *n* = 24). Paranoia did not differ between those with and without Current View ratings (*t* = 0.20, *df* = 35.3, *p* = 0.84). A total of 275 participants completed either the RCADS or the SDQ (mean age = 15.1, SD = 1.75, girls: *n* = 171, boys: *n* = 104, outpatient: *n* = 250, inpatient: *n* = 25). Paranoia was slightly higher in those that completed either measure (mean = 20.3, SD = 18.5) than those who did neither (mean = 15.7, SD = 14.7), although this difference was not significant (*t* = 1.60, *df* = 41.5, *p* = 0.12).

#### Clinician-rated problems

Bivariate associations between paranoia and the presence of each clinician-rated problem are shown in Table 4. The presence of peer relationship problems had the strongest association with paranoia (β *=* 0.64, *p* < 0.001) and explained 11% of the variance in paranoia scores. The second largest association was for self-harm (β *=* 0.55, *p* < 0.001) which accounted for 7% of the variance in paranoia. Similar sized medium associations were also observed for post-traumatic stress symptoms (β *=* 0.54, *p* = 0.001) and a history of abuse/neglect (β *=* 0.50, *p* = 0.013), although only 4% and 2% of the variance in paranoia was explained by these factors, respectively. It was notable that of the 104 patients with at least elevated paranoia, 38 (37%) had clinician-rated trauma (post-traumatic stress or history of abuse/neglect). Depression and social anxiety showed small but significant associations with paranoia that each explained 6% of the variance. Small significant associations accounting for only 4% and 2% of the variance in paranoia were observed for educational difficulties and generalised anxiety, respectively. The presence of ADHD symptoms showed a small negative association that explained 2% of the variance in paranoia scores.

**Table 4.** Associations between paranoia severity and the presence of clinician-rated problems.

Problem type	Clinician rating				Linear regressions			
	Absent		Present		β	95% CI	*p*	*R* ^2^
	*n*	Mean	*n*	Mean				
Social anxiety^a^	79	14.2 (13.7)	189	22.5 (19.4)	**0.45**	[0.23, 0.67]	**<0.001**	**0.06**
Separation anxiety	172	18.5 (17.0)	97	23.1 (20.5)	0.25	[0.00, 0.50]	0.050	0.01
Generalised anxiety	97	16.8 (17.4)	171	21.6 (18.7)	**0.26**	[0.01, 0.51]	**0.042**	**0.02**
OCD	220	20.8 (19.1)	49	17.1 (14.9)	−0.20	[−0.51, 0.11]	0.21	0.01
Panic	187	18.7 (18.2)	84	23.0 (18.7)	0.23	[−0.03, 0.49]	0.078	0.01
Agoraphobia	217	19.6 (18.1)	52	21.8 (19.3)	0.12	[−0.18, 0.43]	0.43	0.00
Depression^a^	75	13.9 (13.2)	197	22.4 (19.5)	**0.46**	[0.25, 0.68]	**<0.001**	**0.06**
Self-harm^a^	143	15.3 (14.3)	129	25.4 (20.8)	**0.55**	[0.31, 0.79]	**<0.001**	**0.07**
Eating problems	222	19.4 (18.6)	50	23.0 (17.3)	0.19	[−0.11, 0.50]	0.21	0.01
Psychosis	254	19.6 (18.1)	17	28.2 (21.6)	0.47	[−0.02, 0.96]	0.061	0.01
Bipolar	246	19.8 (18.6)	26	22.5 (16.4)	0.15	[−0.26, 0.55]	0.48	0.00
PTSD^a^	199	17.7 (16.9)	63	27.5 (20.6)	**0.54**	[0.22, 0.85]	**0.001**	**0.04**
Abuse or neglect^a^	221	18.6 (17.6)	43	27.9 (21.2)	**0.50**	[0.11, 0.89]	**0.013**	**0.02**
Conduct problems	218	19.3 (18.7)	52	23.2 (16.9)	0.22	[−0.09, 0.52]	0.16	0.01
Substance abuse	242	19.4 (18.5)	30	25.4 (16.4)	0.33	[−0.05, 0.71]	0.089	0.01
Emerging PD	208	18.9 (17.8)	62	23.6 (19.5)	0.25	[−0.03, 0.54]	0.080	0.01
Peer difficulties^a^	98	12.5 (13.3)	173	24.2 (19.5)	**0.64**	[0.42, 0.85]	**<0.001**	**0.11**
Family difficulties	111	17.6 (18.4)	157	22.0 (18.3)	0.24	[0.00, 0.48]	0.054	0.01
ADHD^a^	196	21.6 (19.2)	74	15.9 (15.4)	**−0.31**	[−0.55, −0.07]	**0.010**	**0.02**
ASD	172	19.6 (17.6)	93	21.0 (20.2)	0.08	[−0.18, 0.33]	0.56	0.00
Education problems^a^	–	–	–	–	**0.22**	[0.08, 0.36]	**0.002**	**0.04**

β: standardised beta; CI: confidence interval; OCD: obsessive-compulsive disorder; PTSD: post-traumatic stress disorder; PD: personality disorder; ADHD: attention-deficit hyperactivity disorder; ASD: autism spectrum disorder.

Mean paranoia scores shown with standard deviations in parentheses for those with and without each problem. Significant results highlighted in bold.

a Weighted least squares regression used due to heteroscedasticity in residuals.

There was a small-medium association between the presence of clinician-rated psychosis (hallucinations/delusions) and higher paranoia (β = 0.47, *p* = 0.061, *R*^2^ = 0.01). This was not statistically significant, most likely due to limited power with only 17 patients rated as having these symptoms; notably, nine of these (53%) had at least mildly elevated paranoia. Small associations that were not significant (*p* > 0.05) and each accounted for only 1% of the variance in paranoia were observed for substance abuse, emerging personality disorder, separation anxiety, family relationship problems, panic, conduct problems and OCD (Table 4). The associations between paranoia and agoraphobia, extremes of mood, eating problems, and ASD were of a negligible size (β < 0.20) and non-significant (*p* > 0.05).

#### Network analysis

**Figure 1.**
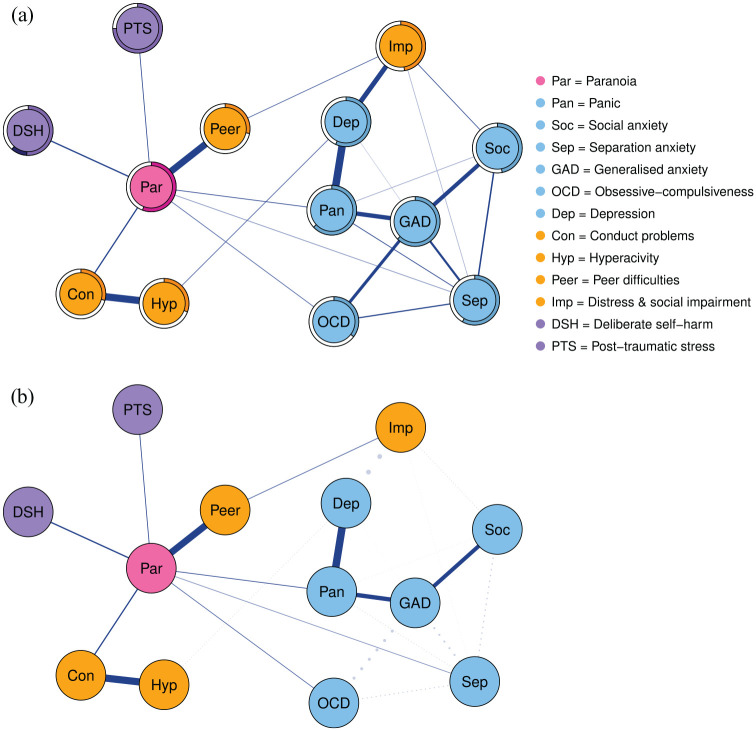
(a) Network analysis of paranoia and other symptoms. Edges indicate positive associations and rings represent node predictability based on neighbouring nodes. Pink, blue, and orange rings (i.e. continuous variables) indicate *R*^2^ values. For binary (i.e. purple) variables, the shaded rings represent the proportion of correct classification, split into the accuracy of the intercept model (purple section) and the additional contribution of connected nodes (dark blue section). (b) Shortest paths from paranoia to all other variables, with dashed lines representing suppressed edges.

The fully estimated network between paranoia, self-report psychological problems and selected clinician-rated symptoms is shown in Figure 1(a) (see supplement for 95% CIs of all edges). Once the contribution of all other variables was controlled, paranoia demonstrated the largest unique relationship with peer difficulties (edge weight = 0.35, 95% CI = [0.22, 0.47]). Figure 1(a) shows paranoia also had a key role in connecting peer difficulties with the rest of the network, with the paths from peer difficulties to four of the anxiety domains, behavioural problems, self-harm, and post-traumatic stress all occurring via paranoia.

Paranoia also demonstrated direct edges with self-harm (edge weight = 0.17, 95% CI = [−0.05,0.38]), conduct problems (edge weight = 0.17, 95% CI = [0.02, 0.31]), panic (edge weight = 0.14, 95% CI = [−0.01, 0.28]), post-traumatic stress (edge weight = 0.14, 95% CI = [−0.07, 0.36]), obsessive compulsiveness (edge weight = 0.11, 95% CI = [−0.03, 0.26]), and separation anxiety (edge weight = 0.08, 95% CI = [−0.05, 0.21]). The edge with peer difficulties was significantly larger than the edges with conduct problems, panic, obsessive-compulsiveness, and separation anxiety (*p* < 0.05) but not self-harm or post-traumatic stress. None of the other edges with paranoia were significantly different in size (*p* > 0.05; supplementary Table S2). A total of 56% of the variance in paranoia was explained by the direct edges with these seven variables (see supplementary Table S2 for predictability values of all nodes). The absence of edges in Figure 1(a) shows that paranoia was conditionally independent from depression, distress/social impairment, hyperactivity, generalised anxiety, and social anxiety, indicating primarily indirect relationships through other variables in the network.

The shortest paths from paranoia to all other variables in Figure 1(b) shows the direct relationship was the dominant pathway between paranoia and all seven variables for which an edge was present. The shortest path network then shows that the fastest route from paranoia to distress/social impairment was via peer difficulties, indicating a mediating role of peer difficulties in this relationship. Potential mediation pathways are also highlighted from paranoia to hyperactivity via conduct problems, and to depression, generalised anxiety, and social anxiety via panic.

Notably, paranoia was the only variable that both self-harm and post-traumatic stress had a unique association with once all other variables were controlled (Figure 1(a)). The normalised accuracy (i.e. predictability) values suggested the single edge with paranoia accounted for 22% of the remaining accuracy of self-harm beyond what was predicted by the intercept model (nCC = 0.22; CC_marg_ = 0.51; CC_total_ = 0.62). Conversely, the edge with paranoia did not lead to any increase in accuracy beyond the intercept model for post-traumatic stress (nCC = 0.00; CC_marg_ = 0.75; CC_total_ = 0.75).

### Paranoia persistence

A total of 105 participants (mean age = 15.1, SD = 1.71, girls: *n* = 75, boys: *n* = 30) agreed to repeat the questionnaires several months later (mean = 21.3 weeks, SD = 6.52). The difference in baseline paranoia between those with follow-up data (mean = 22.6, SD = 19.6) and those without (mean = 18.6, SD = 17.3) was small and not significant (*t* = 1.78, *df* = 190.9, *p* = 0.077).

There was no overall difference in paranoia between baseline (mean = 22.6, SD = 19.6) and follow-up (mean = 23.7, SD = 19.4; *V* = 2296, *p* = 0.73, ES = 0.06). On an individual basis, however, 18/105 participants had significant increases (RCI > 1.96) in paranoia and 16/105 had significant decreases (RCI < −1.96). Of the 46 participants with at least mildly elevated baseline paranoia, 30 had consistently elevated or increasing scores, 5 showed significant reductions that remained in the elevated range, and 11 had significant reductions into the average range.

Linear mixed-effects models showed that, compared to those with low/transient paranoia (*n* = 55), across the two time points, participants with persistent/increasing paranoia (*n* = 50) had consistently higher levels of depression (β = 0.81, 95% CI = [0.45, 1.18], *p* < 0.001), panic (β = 0.75, 95% CI = [0.38, 1.12], *p* < 0.001), social anxiety (β = 0.75, 95% CI = [0.38, 1.11], *p* < 0.001), generalised anxiety (β = 0.74, 95% CI = [0.38, 1.10], *p* < 0.001), separation anxiety (β = 0.64, 95% CI = [0.26, 1.02], *p* = 0.001), peer difficulties (β = 0.63, 95% CI = [0.24, 1.01], *p* = 0.002), conduct problems (β = 0.50, 95% CI = [0.11, 0.90], *p* = 0.014), hyperactivity (β = 0.44, 95% CI = [0.04, 0.84], *p* = 0.032), and distress/social impairment (β = 0.62, 95% CI = [0.23, 1.01], *p* = 0.0026), but not OCD (β = 0.22, 95% CI = [−0.18, 0.63], *p* = 0.28).

There were small paranoia group by time interactions at the threshold for significance for generalised anxiety (β = 0.38, 95% CI = [0.02, 0.74], *p* = 0.043) and social anxiety (β = 0.34, 95% CI = [0.00, 0.68], *p* = 0.052), indicating those with persistent paranoia had somewhat less improvement in these symptoms compared to those with low/transient paranoia. Group by time interactions were negligible and non-significant for all other domains (*p* > 0.05; supplementary Table S4).

## Discussion

The adolescents attending CAMHS were primarily doing so because they had emotional disorders such as anxiety and depression. This was to be expected. However, paranoia was common in these young patients, with several suspicious thoughts occurring in one-third to one-half of the clinical cohort. Over half of patients regularly thought people were lying to them on purpose, over 40% felt scared of what strangers would do to them, and 35% felt unsafe everywhere around people. Overall, 35% reported at least mildly elevated paranoia and 15% reported at least moderate paranoia. Rates of paranoia were approximately double those observed in adolescents from the general population (Bird et al., 2019). Previous findings that adolescent girls, compared to boys, may be especially likely to report suspicious thinking were replicated (Bird et al., 2019; Ronald et al., 2014). Although traditionally conceptualised as a symptom of psychotic disorders, paranoia in this adolescent sample primarily occurred alongside common mental health problems and only a minority had suspected psychosis. Although limited in size, the available follow-up data indicated that the paranoia was often persistent. Yet paranoia may well be overlooked: only one participant had the presence of paranoia recorded in their clinical notes.

Paranoid thinking in the adolescent patients was associated with a wide range of clinician-rated problems including anxiety, depression, trauma, self-harm, peer relationship, and educational difficulties. Paranoia in CAMHS patients may therefore be expected to present in the context of emotional problems, adverse life experiences, and impaired social functioning. It may also be particularly common in young people who self-harm: elevated paranoia was present in almost half of patients for whom emotion dysregulation, self-harm, or suicidality was a primary reason for accessing services. Network analysis also showed that once all other variables were controlled, the presence of self-harm was solely associated with paranoia, with this edge contributing to 22% of the predictability of self-harm (beyond the intercept model). This relationship is consistent with findings from the adult literature (Freeman et al., 2019b) and evidence that self-harm is associated with psychotic experiences in general in adolescents (Hielscher et al., 2019; Martin et al., 2015). The co-occurrence of paranoia with so many different psychiatric symptoms could also be an indicator of more severe presentations, with adolescents who report persistent paranoia having greater levels of symptoms and social impairments over time.

Consistent with a cognitive conceptualisation of paranoia as an unfounded threat belief (Freeman, 2016), network analyses showed paranoia had unique associations with anxiety symptoms, especially panic. The network analysis further demonstrated a relationship between paranoia and post-traumatic stress symptoms. Once all other variables were controlled, the presence of post-traumatic stress symptoms was solely related to paranoia. This relationship is consistent with evidence that negative interpersonal experiences contribute to the development of paranoia (Freeman et al., 2013; Shevlin et al., 2015). It is important to emphasise, however, that justified fears of harm in relation to ongoing bullying or abuse is not paranoia (a term that only applies to unfounded ideas). Paranoia in those with adversity occurs when their concerns generalise excessively beyond specific experiences to the point they become clearly unfounded (e.g. when an individual with past bullying develops a persistent concern that people are conspiring to humiliate them and interprets friendliness from others as a trick). Although several mechanisms driving this generalisation are likely, one proposal is that negative experiences lead to learned beliefs about other people (i.e. as threatening) and the self (i.e. as vulnerable) upon which paranoia flourishes (Freeman, 2016). Paranoia can be an understandable protective response to a dangerous world, though this does not mean it is inevitable or that it is without negative consequences. But our findings also show paranoia is certainly not confined to traumatised youth: the trauma variables only accounted for a very small amount of the variance in paranoia and almost two-thirds of patients with paranoia did not have a (clinician-rated) history of trauma.

Arguably one of the most important findings from the study is a close relationship between paranoia and peer relationship difficulties. This association was the strongest of all those assessed from both clinicians and patients, even after controlling for the influence of all other variables in the network. Although the relationship will undoubtedly be bidirectional to a degree, our previous work using a Bayesian approach to causal discovery found adolescent peer difficulties are more likely to be influenced by paranoia than vice versa (Bird et al., 2019). This pathway is plausible, as the ability to trust is necessary for relationships, whereas fear of other people will make it difficult to socialise and make friends. We also found the most common pathway from emotional and behavioural problems to peer difficulties occurred via paranoia, suggesting paranoia may be a common route to impairments in adolescent peer relationships. At an age when peer acceptance is most highly valued (Somerville, 2013), the potential impact on friendships is likely to be a substantial cause of distress for young people. In line with this, peer difficulties were the mediating link connecting paranoia and the overall distress and functional impact of young people’s problems.

### Limitations

The study has several limitations. First, the sample was not a fully representative cohort. It was not possible to invite all patients accessing participating services to take part, since services could not be covered by the research team all the time. However, attempts were made to minimise sampling bias by inviting patients to take part regardless of their reason for accessing services or clinical diagnosis. The cohort also included a higher proportion of girls than boys, although this may be representative of CAMHS given the higher rates of common mental health problems in adolescent girls (NHS Digital, 2018). Nevertheless, the pattern of associations between paranoia and other variables could be influenced by gender, and, as a result, the network structure may have biased understanding towards girls. However, there is a lack of clear evidence showing the relationships between paranoia and causal factors differ by gender. Another notable source of sampling bias was the primarily affluent catchment areas for the services included with a local demographic of mostly White British individuals. As experiences such as racism and child adversity are likely to contribute to the development of paranoia (Bentall et al., 2012; Shaikh et al., 2016), clinical levels of paranoia in youth may differ by locality.

A strength of this study was the ability to compare the prevalence of paranoia in CAMHS patients with a representative general population sample of adolescents using the same measure. This was not a perfect comparison, however, as the general population sample were slightly younger than the patients in this study. But as age was not associated with paranoia in either sample, the effect of this difference on the comparison is likely to be minimal. Another limitation was that aside from the B-CAP, our other measures were missing for approximately 10% of participants. This reflected the reality of routine measurement in CAMHS where clinical pressures could prevent clinicians from completing the Current View and patients sometimes left before completing all questionnaires. Notably, as follow-up questionnaires were collected as an optional second stage of the study, only a third of the sample provided longitudinal data. Planned prospective studies examining paranoia in representative clinical samples will be needed to understand fully the relationship over time with other mental health problems.

It must also be acknowledged that a degree of measurement error is likely in self-report measures of paranoia. It is not possible in self-report questionnaires to determine if concerns about intended harm from others are unfounded. However, the B-CAP has shown good construct validity as a measure of unfounded ideation, with evidence that scores are distinct from bullying and are associated with adolescents’ ratings that their fears of others are excessive (Bird et al., 2019, 2020). Evidence also shows that, in general, self-report paranoia questionnaires predict genuine paranoid ideation in controlled virtual reality settings (Freeman et al., 2010, 2014). Nevertheless, clinical interview validation of paranoia in young people accessing CAMHS would be beneficial.

Overall, this study highlights paranoia as a common, potentially clinically important, and overlooked problem in young people who are accessing mental health services. Greater awareness by clinicians of paranoia in patients attending CAMHS may be required. The use of validated tools such as the B-CAP may be helpful for clinicians to identify paranoia within young people’s broader clinical presentation and monitor change. Targeted intervention for paranoia, suitably adapted for this age group, may then be appropriate to help young people feel safer in their daily lives.

## Supplemental Material

sj-pdf-1-anp-10.1177_0004867420981416 – Supplemental material for Paranoia in patients attending child and adolescent mental health services Click here for additional data file. Supplemental material, sj-pdf-1-anp-10.1177_0004867420981416 for Paranoia in patients attending child and adolescent mental health services by Jessica C Bird, Emma C Fergusson, Miriam Kirkham, Christina Shearn, Ashley-Louise Teale, Lydia Carr, Hannah J Stratford, Antony C James, Felicity Waite and Daniel Freeman in Australian & New Zealand Journal of Psychiatry
