# Supplementary material for: Paranoia in patients attending child and adolescent mental health services
Source: Aust N Z J Psychiatry. 2021 Jan 10;55(12):1166–77. doi: 10.1177/0004867420981416 (PMC8649424; doi:10.1177/0004867420981416)
Supplement: sj-pdf-1-anp-10.1177_0004867420981416 – Supplemental material for Paranoia in patients attending child and adolescent mental health services [file sj-pdf-1-anp-10.1177_0004867420981416.pdf]

SUPPLEMENTARY MATERIALS

1. Correlations

Table S1. Simple correlations between paranoia and self-report symptom domains

	n	*r*	*p*
Panic	269	0.55	<0.001
Peer difficulties	268	0.51	<0.001
Separation anxiety	269	0.50	<0.001
Generalised anxiety	270	0.49	<0.001
Social anxiety	267	0.46	<0.001
OCD	269	0.44	<0.001
Depression	271	0.43	<0.001
Distress/impairment	261	0.38	<0.001
Conduct problems	271	0.27	<0.001
Hyperactivity	268	0.21	<0.001

2. Node predictability

Table S2. Predictability of each node in the network based on neighbouring nodes. R^2 indicate the proportion of variable explained for continuous variables. For binary variables, CC_{total} values indicate the total accuracy, CC_{marg} represent the accuracy of the marginal intercept model, and nCC represents the predictability beyond the intercept model.

**Node**	**R^2**	**CC_{total}**	**CC_{marg}**	**nCC**
Paranoia	0.56	/	/	/
Panic	0.63	/	/	/
Social anxiety	0.47	/	/	/
Separation anxiety	0.58	/	/	/
Generalised anxiety	0.63	/	/	/
Obsessive compulsiveness	0.35	/	/	/
Depression	0.57	/	/	/
Conduct problems	0.29	/	/	/
Hyperactivity	0.31	/	/	/
Peer difficulties	0.28	/	/	/
Distress/impairment	0.48	/	/	/
Self-harm	/	0.62	0.51	0.22
Post-traumatic stress	/	0.75	0.75	0.00

2. Confidence intervals of partial correlation edge weights

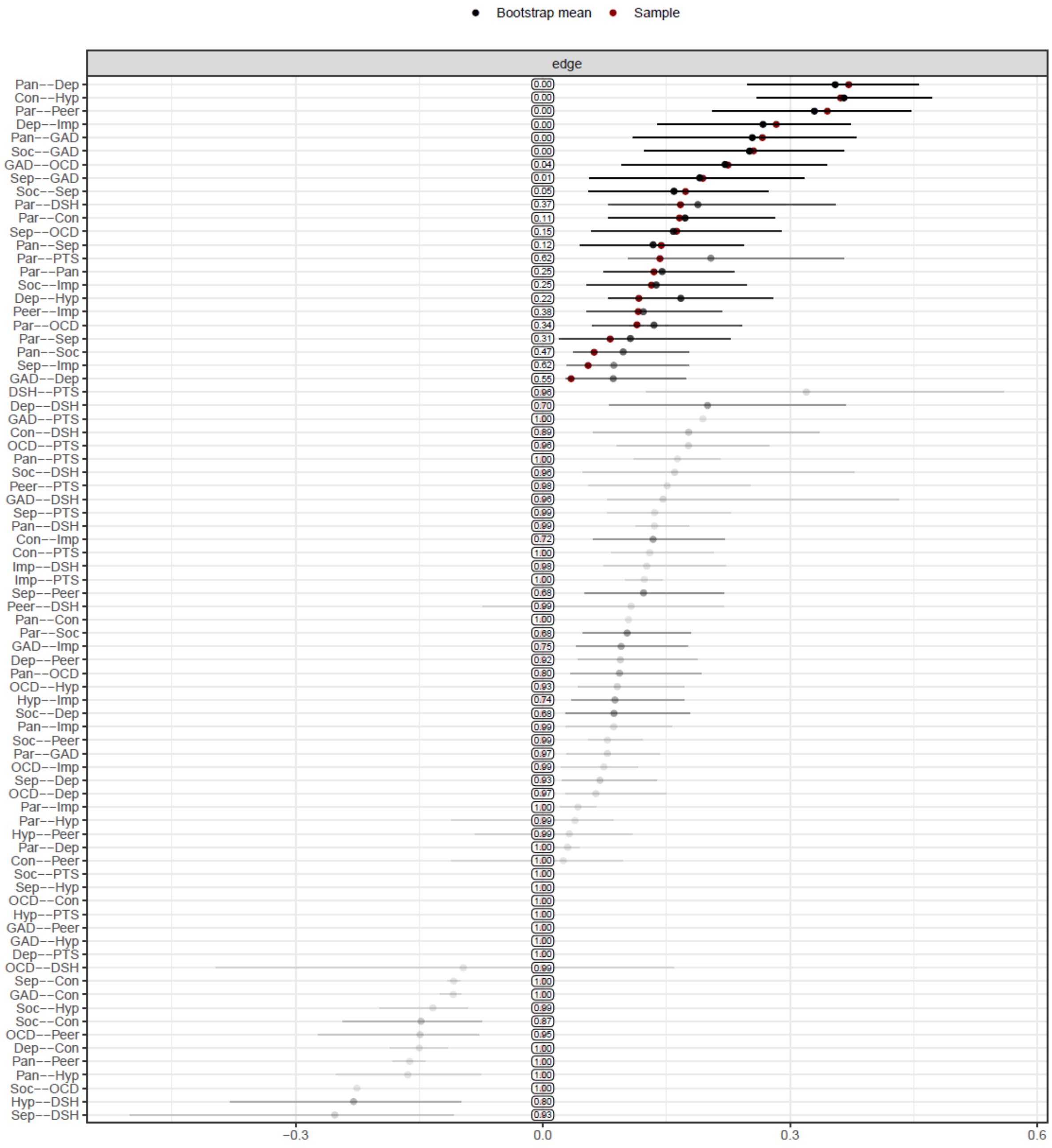

Figure S1. Edge weights for all edges in the network with bootstrapped 95% confidence intervals. The proportion of bootstrapped cases that were set to zero are shown for each edge. Grey lines indicate a zero-partial correlation (i.e. lack of an edge) between those two variables.

3. Bootstrap edge difference test

Table S3. Bootstrap edge difference test comparing the size of the edges connecting with paranoia.

Node 1	Node 2	Lower CI	Upper CI	$p < 0.05$
**Peers**	Panic	-0.41	-0.04	TRUE
	Conduct problems	-0.39	0.00	TRUE
	Obsessive compulsiveness	-0.41	-0.05	TRUE
	Social anxiety	-0.43	-0.13	TRUE
	Separation anxiety	-0.41	-0.07	TRUE
	Generalised anxiety	-0.45	-0.19	TRUE
	Self-harm	-0.41	0.03	FALSE
	Post-traumatic stress	-0.43	0.04	FALSE
**Panic**	Conduct problems	-0.19	0.24	FALSE
	Obsessive compulsiveness	-0.20	0.20	FALSE
	Social anxiety	-0.22	0.12	FALSE
	Separation anxiety	-0.20	0.18	FALSE
	Generalised anxiety	-0.23	0.00	FALSE
	Self-harm	-0.21	0.27	FALSE
	Post-traumatic stress	-0.21	0.26	FALSE
**Conduct**	OCD	-0.26	0.16	FALSE
	Social anxiety	-0.27	0.07	FALSE
	Separation anxiety	-0.25	0.14	FALSE
	Generalised anxiety	-0.28	0.00	FALSE
	Self-harm	-0.25	0.23	FALSE
	Post-traumatic stress	-0.27	0.21	FALSE
**OCD**	Social anxiety	-0.23	0.14	FALSE
	Separation anxiety	-0.21	0.20	FALSE
	Generalised anxiety	-0.23	0.00	FALSE
	Self-harm	-0.18	0.28	FALSE
	Post-traumatic stress	-0.20	0.26	FALSE
**Social anxiety**	Separation anxiety	-0.13	0.21	FALSE
	Generalised anxiety	-0.16	0.00	FALSE
	Self-harm	-0.13	0.31	FALSE
	Post-traumatic stress	-0.15	0.32	FALSE
**Separation anxiety**	Generalised anxiety	-0.21	0.00	FALSE
	Self-harm	-0.18	0.28	FALSE
	Post-traumatic stress	-0.20	0.29	FALSE
**Generalised anxiety**	Self-harm	0.00	0.33	FALSE
	Post-traumatic stress	0.00	0.32	FALSE
**Self-harm**	Post-traumatic stress	-0.29	0.25	FALSE

4. Follow up descriptives

Table S3. Descriptive statistics for follow up data. Standard deviations in parentheses

**Measure**	**Time 1**		**Time 2**		**Difference**		
	n	Score	*n*	Score	Mean difference	Effect size	*p*
Paranoia	105	22.6 (19.6)	105	23.7 (19.4)	1.10 (16.6)	0.06	0.73 [a]
Depression	94	17.1 (6.78)	101	15.4 (6.78)	-1.56 (5.63)	0.26	0.014 [a]
Panic	93	11.6 (7.33)	100	10.6 (7.02)	-0.75 (5.25)	0.14	0.21 [a]
Social anxiety	92	17.5 (6.76)	100	15.9 (7.06)	-1.43 (5.55)	0.24	0.019
Separation anxiety	92	7.58 (4.84)	100	7.03 (4.70)	-0.43 (3.33)	0.11	0.15 [a]
OCD	92	6.91 (4.37)	100	5.73 (4.46)	-0.99 (3.93)	0.27	0.018 [a]
GAD	93	10.1 (4.63)	100	9.27 (4.72)	-0.72 (4.12)	0.19	0.24 [a]
Peer difficulties	93	3.78 (2.21)	99	3.80 (2.09)	0.02 (1.66)	0.01	0.69 [a]
Conduct	94	3.21 (2.14)	99	3.05 (2.12)	-0.03 (1.67)	0.08	0.40 [a]
Hyperactivity	93	6.18 (2.35)	99	6.22 (2.53)	0.22 (1.92)	0.02	0.30
Distress/impairment	90	9.11 (3.33)	99	7.97 (4.20)	-0.83 (3.63)	0.34	0.052 [a]

[a] Non-parametric Wilcoxon test used as the difference scores were not normally distributed ($p<0.05$)

5. Repeated measures mixed effects model

Table S4. Mixed-effects models of the relationship between paranoia trajectory group and symptom domains over time. Mean paranoia scores are shown with standard deviation in parentheses, and standardised beta coefficients are shown with 95% confidence intervals.

Measure	Paranoia scores		Group effect		Group*Time effect	
	Low	Persistent	β	p	β	p
Depression						
Time 1:	14.5 (6.06)	20.0 (6.42)	0.81 (0.45,1.18)	<0.001	0.12 (-0.22,0.47)	0.49
Time 2:	12.1 (5.77)	18.9 (6.00)				
Panic						
Time 1	9.10 (6.76)	14.5 (6.96)	0.75 (0.38,1.12)	<0.001	0.22 (-0.08,0.52)	0.16
Time 2	7.17 (5.70)	14.4 (6.40)				
Social anxiety						
Time 1	15.1 (7.20)	20.2 (5.03)	0.75 (0.38,1.11)	<0.001	0.34 (0.00,0.68)	0.052
Time 2	12.0 (6.22)	20.0 (5.40)				
Separation anxiety						
Time 1	6.14 (4.82)	9.21 (4.37)	0.64 (0.26,1.02)	0.001	0.25 (-0.04,0.54)	0.092
Time 2	4.85 (3.85)	9.40 (4.41)				
OCD						
Time 1	6.45 (4.57)	7.44 (4.11)	0.22 (-0.18,0.63)	0.28	0.23 (-0.14,0.60)	0.22
Time 2	4.69 (4.51)	6.85 (4.17)				
Generalised anxiety						
Time 1	8.51 (4.85)	12.0 (3.62)	0.74 (0.38,1.10)	<0.001	0.38 (0.02,0.74)	0.043
Time 2	6.56 (4.13)	12.2 (3.40)				
Peer difficulties						
Time 1	3.13 (1.87)	4.46 (2.34)	0.63 (0.24,1.01)	0.002	0.10 (-0.23,0.42)	0.57
Time 2	2.96 (1.60)	4.69 (2.20)				
Conduct problems						
Time 1	2.69 (1.81)	3.76 (2.33)	0.50 (0.11,0.90)	0.014	0.08 (-0.25,0.40)	0.65
Time 2	2.45 (1.94)	3.69 (2.14)				
Hyperactivity						
Time 1	5.66 (2.29)	6.72 (2.33)	0.44 (0.04,0.84)	0.032	-0.01 (-0.35,0.33)	0.96
Time 2	5.75 (2.77)	6.73 (2.15)				
Distress/impairment						
Time 1	8.00 (3.64)	10.3 (2.46)	0.62 (0.23-1.01)	0.002	0.09 (-0.32-0.50)	0.67
Time 2	6.45 (4.09)	9.58 (3.71)				
